# Site-Specific Competitive Kinase Inhibitor Target Profiling Using Phosphonate Affinity Tags

**DOI:** 10.1016/j.mcpro.2025.100906

**Published:** 2025-01-16

**Authors:** Wouter van Bergen, Anneroos E. Nederstigt, Albert J.R. Heck, Marc P. Baggelaar

**Affiliations:** 1 Biomolecular Mass Spectrometry and Proteomics, Bijvoet Center for Biomolecular Research and Utrecht Institute for Pharmaceutical Sciences, University of Utrecht, Utrecht, CH, The Netherlands; 2 Netherlands Proteomics Center, Utrecht, CH, The Netherlands

**Keywords:** kinase inhibitor, drug-target, inhibitor selectivity, chemical proteomics, activity-based protein profiling

## Abstract

Protein kinases are prime targets for drug development due to their involvement in various cancers. However, selective inhibition of kinases, while avoiding off-target effects remains a significant challenge for the development of protein kinase inhibitors. Activity-based protein profiling (ABPP), in combination with pan-kinase activity-based probes (ABPs) and mass spectrometry–based proteomics, enables the identification of kinase drug targets. Here, we extend existing ABPP strategies for kinase profiling with a site-specific analysis, allowing for protein kinase inhibitor target engagement profiling with amino acid specificity. The site-specific approach involves highly efficient enrichment of ABP-labeled peptides, resulting in a less complex peptide matrix, straightforward data analysis, and the screening of over ∼100 kinase active sites in a single LC-MS analysis. The complementary use of both trypsin and pepsin in parallel to generate the ABP-labeled peptides considerably expanded the coverage of kinases and pinpoint the exact binding sites. Using the site-specific strategy to examine the on- and off-targets of the Ephrin receptor (Eph) B4 inhibitor NVP-BHG712 showed binding to EphA2 with an IC_50_ of 17 nM and EphB4 with an IC_50_ of 20 nM. Next to the known targets, EphA2 and EphB4, NVP-BHG712 bound to the discoidin domain-containing receptor 1 with an IC_50_ of 2.1 nM, suggesting that a discoidin domain-containing receptor 1–targeting regio-isomer of NVP-BHG712 was used. The promiscuity of XO44 toward ATP-binding pockets on nonkinase proteins facilitated the screening of additional off-target sites, revealing inosine-5′-monophosphate dehydrogenase 2 as a putative off-target. Expanding the search to other amino acids revealed that XO44, in addition to 745 lysines, also covalently linked 715 tyrosines, which significantly expands the competitive ABPP search space and highlights the added value of the site-specific method. Therefore, the presented approach, which can be fully automated with liquid handling platforms, provides a straightforward, valuable new approach for competitive site-specific kinase inhibitor target profiling.

## Highlights

• Phosphonate handles enable automated enrichment of ABP-bound peptides.
• Competitive site-specific ABPP profiles drug targets and off-targets simultaneously.
• Site-specific ABPP distinguishes multiple binding sites within proteins.
• Site-specific ABPP reduces false positives originating from co-eluted proteins.

## Graphical Abstract

**Figure:**
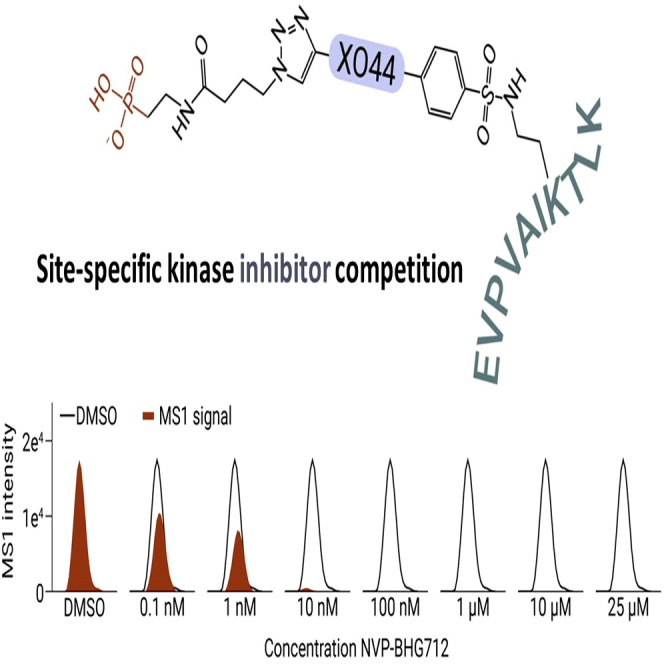

## In Brief

Protein kinases are prime targets in cancer drug development, yet achieving selective inhibition while avoiding off-target effects is challenging. Activity-based protein profiling (ABPP) with pan-kinase probes and mass spectrometry enables efficient identification of kinase drug targets in native proteomes. Our competitive, site-specific ABPP approach profiles over 100 kinase active sites simultaneously and allows selectivity profiling against more than 300 sites in nonkinase proteins. This method includes automated enrichment and, unlike protein-centric methods, can differentiate between multiple binding sites within single proteins.

Protein kinases are validated drug targets, especially in cancer (1, 2, 3). The human kinome contains 557 protein kinases that regulate cellular signaling through phosphorylation, inducing changes in protein function, structure, localization, and interactions (4). Phosphorylation affects approximately 75% of all human proteins, underscoring the pivotal role of protein kinases in virtually every cellular process (5). Consequently, aberrant kinase function often results in diseases like cancer (6). Protein kinase inhibitors, small molecule drugs inhibiting kinase activity, are used therapeutically, for instance, in targeting erythropoietin-producing hepatocellular (Eph) tyrosine-kinase receptors in glioblastoma, the epidermal growth factor receptor in nonsmall cell lung cancer, and the Bruton tyrosine kinase in B-cell lymphoma (7, 8, 9, 10, 11).

Despite significant progress in protein kinase inhibitor development, the need for new, efficient, and safe protein kinase inhibitors persists in addressing various types of cancer (12, 13). High attrition rates, mainly due to toxicity induced by off-target events, reaching up to 90%, contribute to numerous unsuccessful drug development endeavors (3, 14). The development of selective kinase inhibitors is challenging because many of the current drugs target ATP-binding pockets in the protein kinase active site, which are conserved across numerous proteins in the proteome, resulting in undesired off-target binding (15, 16). Understanding the precise target scope of a protein kinase inhibitor, ideally in the context of living cells, can facilitate the analysis and prediction of the safety and efficiency of candidate protein kinase inhibitors.

High-throughput screening for protein kinase inhibitors often relies on the enzymatic activity of isolated kinases or kinase domains *in vitro*. However, the interactions between small molecule drugs and isolated kinase (domains) may not necessarily mirror the *in vivo* situation, as the cellular context can affect protein conformation, activity, and localization of these kinases (17, 18). Chemical proteomics enables the identification and quantification of inhibitor binding to endogenous protein kinases in complex environments (19). Competition-based assays, such as bead-immobilized kinase inhibitors (“kinobeads”) and irreversible ATP-(desthio)biotin probes, efficiently capture kinases for liquid chromatography-mass spectrometry (LC-MS) analysis (20, 21, 22, 23, 24, 25, 26). While these tools offer excellent screening abilities by interrogating up to 200 endogenous kinases, they lack cell permeability. Consequently, kinobeads or ATP-based probes can only be applied to lysates, which may contain different activation states of protein kinases compared to endogenous cellular environments. Recently, Zhao *et al.* developed XO44, a cell-permeable 2-step activity-based probe (ABP) that targets a broad range of protein kinases (16). Mass spectrometry proteomics analysis, following biotin-based enrichment of ABP-bound proteins, resulted in the detection of 120 kinases from a single cell line. Site-specific analysis of these ABP labeling sites would allow accurate analysis of the endogenous kinome while reducing false positive identifications. While an XO44 derivative containing a sulfonyl−triazole reactive group instead of a sulfonyl fluoride (KY-26) in combination with a desthiobiotin enrichment enabled the site-specific identification of ABP–kinase interactions, the coverage remained limited to 23 protein kinases (27). The strong binding affinity between biotin and streptavidin offers a highly efficient enrichment strategy. However, this same strong interaction poses challenges in the elution of biotinylated peptides from streptavidin resin. Recently, we introduced PhosID-ABPP, a powerful site-specific ABPP strategy (28). We hypothesized that this strategy, in combination with the profiling abilities of the XO44, allows a more robust and complete site-specific analysis of the endogenous kinome.

By integrating PhosID-ABPP with the ABP XO44, we successfully identified over 1000 ABP-binding sites, encompassing approximately 100 protein kinases. In a competitive fashion, this broad platform enables site-specific target profiling of kinase inhibitors in intact cells.

## Experimental Procedures

### Cell Culture

A549 cells (CCL-185, ATCC) with a passage number below 20 were cultured in growth medium [(Dulbecco’s modified eagle medium (Gibco) supplemented with 10% fetal bovine serum (HyClone GE) and 100 units/ml penicillin-streptomycin (Gibco)]. Cells were grown in a humidified atmosphere with 5% CO_2_ at 37 °C in T175 flasks (Greiner). Cells were split twice weekly by washing with Dulbecco’s phosphate buffered saline (DPBS, Lonza) and were treated with 0.05% Trypsin-EDTA (Gibco) for cell detachment. After detachment, trypsin was quenched by adding growth medium. 1/10 of the cell suspension was taken and grown with fresh growth medium in a new T175 flask.

### Activity-Based Protein Profiling Experiment in Cell Culture

1e^6^ cells were plated in 15 cm plates (Greiner) 3 days before treatment and kept in a humidified atmosphere with 5% CO_2_ at 37 °C. The growth medium was replaced by 10 ml treatment [DMEM with 0.5% FBS and 100 units/ml PS supplemented with the corresponding concentration of NVP-BHG712 (Sigma-Aldrich) or DMSO] and incubated at 37 °C, 5% CO_2_ for 3 h. Then, 10 μl 25 mM XO44 (PF-6808472, Sigma-Aldrich) was added directly to the medium and swirled to enable dissolution. Subsequently, the cells were incubated at 37 °C, 5% CO_2_ for 4 h. The samples for Supplemental Fig. S1 were generated by directly incubating with 25 μM XO44-containing growth medium for 4 h.

### Cell Harvest

After treatment, the cells were washed with ice-cold DPBS and harvested using a cell scraper in 1 ml ice-cold DPBS. Then, the cell suspension was spun down at 2000*g* for 1 min, and the supernatant was aspirated. The cell pellet was snap-frozen in liquid nitrogen and stored at −80 °C for later use.

### Cell Lysis

Cell pellets were lysed in 500 μl lysis buffer per 15 cm plate of cells, consisting of 50 mM Hepes (Sigma-Aldrich, pH 7.5), 0.5% NP-40 (Applichem), 0.2% SDS (Gen-Apex), 2 mM MgCl_2_ (Sigma-Aldrich), 10 mM NaCl (Merck), 1× protease inhibitor cocktail (Roche), and 0.5 μl/ml Benzonase (Millipore). Cell lysates were incubated at room temperature for 15 min to allow DNA cleavage. Cell debris and DNA were spun down for 30 min at 20,567*g* at 16 °C. The supernatant was collected, and the protein concentration was determined by a bicinchoninic acid assay (Thermo Fisher Scientific).

### Bioorthogonal Chemistry Reactions for Proteomics

The copper(I)-catalyzed azide-alkyne cycloaddition (CuAAC) was performed on cellular lysates in 2 M urea (Merck) in 1× 50 mM Hepes (pH 7.5). CuAAC components were added in the following order: 5 mM tris(3-hydroxypropyltriazolylmethyl)amine (Lumiprobe), 2.5 mM CuSO_4_ 5·H_2_O (Sigma-Aldrich), 500 μM phosphonate-azide (prepared as described in van Bergen *et al*., 2023 (28)), and 25 mM sodium ascorbate (Sigma-Aldrich). Samples were incubated for 2 h at room temperature while rotating. Methanol–chloroform precipitation was performed to remove the CuAAC components, and the air-dried pellets were resuspended in 8 M urea and sonicated in a bioruptor (Diagenode) with the high amplitude setting for 10 min with cycles of 30 s on and 30 s off.

### Sample Processing for Digestion

Clicked and dissolved protein samples were diluted to 4M urea with 50 mM ammonium bicarbonate (pH 8, AmBic, Sigma-Aldrich). The proteins were reduced with 4 mM DTT (Sigma-Aldrich) for 60 minutes at room temperature and alkylated in the dark using 8 mM iodoacetamide (Sigma-Aldrich) for 30 min. Residual iodoacetamide was quenched by adding DTT to a final concentration of 4 mM.

Next, samples were diluted 2× with 50 mM AmBic and digested with LysC (1:75 enzyme to protein ratio, Wako) for 4 h at 37 °C. Finally, proteins were digested overnight using Trypsin (1:50, enzyme to protein ratio, Sigma-Aldrich) at 37 °C. Proteolysis with chymotrypsin was performed similarly to the trypsin digestion, only replacing both proteolytic enzymes with α-chymotrypsin (bovine, 1:75, enzyme to protein ratio, Sigma-Aldrich) overnight at 37 °C. Digested material was desalted using 3 cc C18 Seppak cartridges (Waters) and air dried using a vacuum centrifuge.

For the digestion with pepsin, protease incubation (Porcine, 1:50, enzyme to protein ratio, Sigma-Aldrich) was performed for 4 h at 37 °C in 40 mM HCl (pH 2). Digested material was directly desalted using 3 cc C18 Seppak cartridges and air dried using a vacuum centrifuge.

### Dephosphorylation

Samples were dephosphorylated prior to immobilized metal affinity chromatography (IMAC) enrichment. Desalted peptides were dissolved in 1× rCutSmart buffer (pH 8, New England BioLabs) and incubated with 50 units of alkaline phosphatase (calf intestinal, QuickCIP, New England BioLabs) overnight at 37 °C while shaking. After dephosphorylation, all peptides were again desalted using 3 cc C18 Seppak cartridges and air-dried using a vacuum centrifuge.

### Automated Fe^3+^-IMAC Enrichment

Probe-phosphonate–labeled peptides were enriched using Fe(III)-NTA 5 μl (Agilent Technologies) in an automated fashion by the AssayMAP Bravo Platform (Agilent Technologies). Fe(III)-NTA (nitrilotriacetic acid) cartridges were primed at a flow rate of 100 μl/min with 250 μl of priming buffer [0.1% TFA, 99.9% acetonitrile (ACN)] and equilibrated at a flow rate of 50 μl/min with 250 μl of loading buffer (0.1% TFA, 80% ACN). The flow through was collected into a separate plate. Dried peptides were dissolved in 200 μl of loading buffer and loaded at a 2 μl/min flow rate onto the cartridge. Columns were washed with 250 μl of loading buffer at a flow rate of 20 μl/min, and the phosphonate-labeled peptides were eluted with 35 μl of ammonia (10%) at a flow rate of 5 μl/min directly into 35 μl of formic acid (10%). Flowthroughs and elutions were air-dried afterward and stored at −20 °C.

### Experimental Design and Statistical Rationale

All dose-response analysis experiments were performed in triplicate (n = 3) biological replicates to control for variability in the workflows and ensure the reproducibility of the results. We employed seven different inhibitor concentrations and a DMSO control in the dose-response assays to establish both the top and bottom plateaus, enabling accurate determination of IC_50_ values for specific drug-protein combinations.

### Liquid Chromatography-Mass Spectrometry

Prior to analysis, dried peptides were dissolved in 20 μl of 2% formic acid supplemented with 20 mM citric acid (Sigma-Aldrich). Subsequently, the IMAC-enriched peptides were injected and separated by an Ultimate 3000 nanoUHPLC system (Thermo Fisher Scientific) equipped with a PepSep column (25 cm × 150 μm, 1.5 μm, C18; Bruker Daltonics) heated to 50 °C by an external column oven (Sonation). The peptides were separated in a 71 min gradient (7.1 min 3% B, 73.1 min 25% B, 78 min 45% B) at a flow rate of 800 nl/min using 0.1% formic acid in Milli Q as solvent A and 0.1% formic acid in ACN as solvent B. The LC system was coupled to a trapped ion mobility quadrupole time-of-flight mass spectrometer timsTOF HT (Bruker Daltonics) *via* a nanoelectrospray ion source CaptiveSpray (Bruker Daltonics).

Data acquisition on the timsTOF HT was performed using TIMSControl 4.0.5.0 and Compass HyStar 6.0.30.0 (Bruker Daltonics) starting from the DDA-PASEF method optimized for standard proteomics. This method utilized a capillary voltage of 1300 V, nebulizer dry gas flow rate of 3.0 l/min at 180 °C, MS/MS target intensity of 20,000 counts, and dynamic exclusion of precursor release after 0.4 min. Singly charged peptides were excluded by an active inclusion/exclusion polygon filter applied within the ion mobility over *m/z* heatmap. Data were acquired in a 100 to 1700 m/z range with 10 PASEF ramps (100 ms accumulation/ramp) with a total cycle time of 1.17 s. The TIMS range was set to 0.7 to 1.3 Vs/cm^2^, precursor Intensity threshold to 1500 counts, the linearly interpolated ion mobility–dependent collision energy to 20 eV at 0.6 Vs/cm^2^ 60 eV at 1.6 Vs/cm^2^, and precursor charge restriction to 3+ to 5+.

### Database Search and Analysis

LC-MS/MS run files were searched against the human (20,375 entries) SwissProt database (version September 2020) using Fragpipe v20.0 with MSFragger 3.8, IonQuant 1.9.8, and Philosopher 5.0.0 search engine using the default settings (29). The integrated Fragpipe contaminant database was used to filter out contaminants. The cleavage site was set to K/R for trypsin, F/L/Y/W for chymotrypsin, and nonspecific for pepsin. A peptide length between 5 and 30 was allowed. Up to two missed cleavages were allowed for trypsin and no missed cleavages were defined for pepsin as a nonspecific search was performed for pepsin digests. Carbamidomethylation of cysteines was set as fixed modification. Oxidation of methionine, acetylation of the protein N-terminus, and XO44-phosphonate (754.2532) on lysine were set as variable modifications. In addition to lysine, XO44-phosphonate (754.2532) was also set as a variable modification on tyrosine residues in searches to monitor if XO44 binds to other amino acid residues within proteins. All modifications were used in the first search. Precursor and fragment mass tolerance were set to 20 and 50 ppm, respectively. The false discovery rate for peptide-spectrum matches and proteins was set to 1% using a target-decoy approach. MaxLFQ with match-between-runs and normalization was used for quantification.

### Data Analysis, Statistical Analysis, and Visualization

The “combined_modified_peptides.tsv” tables were used for the quantitative analysis of XO44-binding sites in RStudio 2023.6.2.561 (30). XO44-bound peptides were filtered for presence in at least two replicates in DMSO samples and were summed and clustered to each XO44-binding site. The curve-fitting, IC_50_ calculation, and visualization of graphs were done in GraphPad Prism 10.1.2. Venn diagrams and upset plots were created using R packages Eulerr and UpsetR, respectively (31, 32). The phylogenetic clustering and tree visualization were performed with Clustal omega and the interactive tree of life (33, 34). MS1 chromatography traces and MS/MS spectra were extracted using Skyline-daily 23.1.1.353 (35). The figures were finally compiled and visualized using Adobe Illustrator 28.1.

## Results and Discussion

### Protease Complementarity Extends the Scope of PhosID-ABPP–Assisted Site-Specific Kinome Profiling

**Fig. 1:**
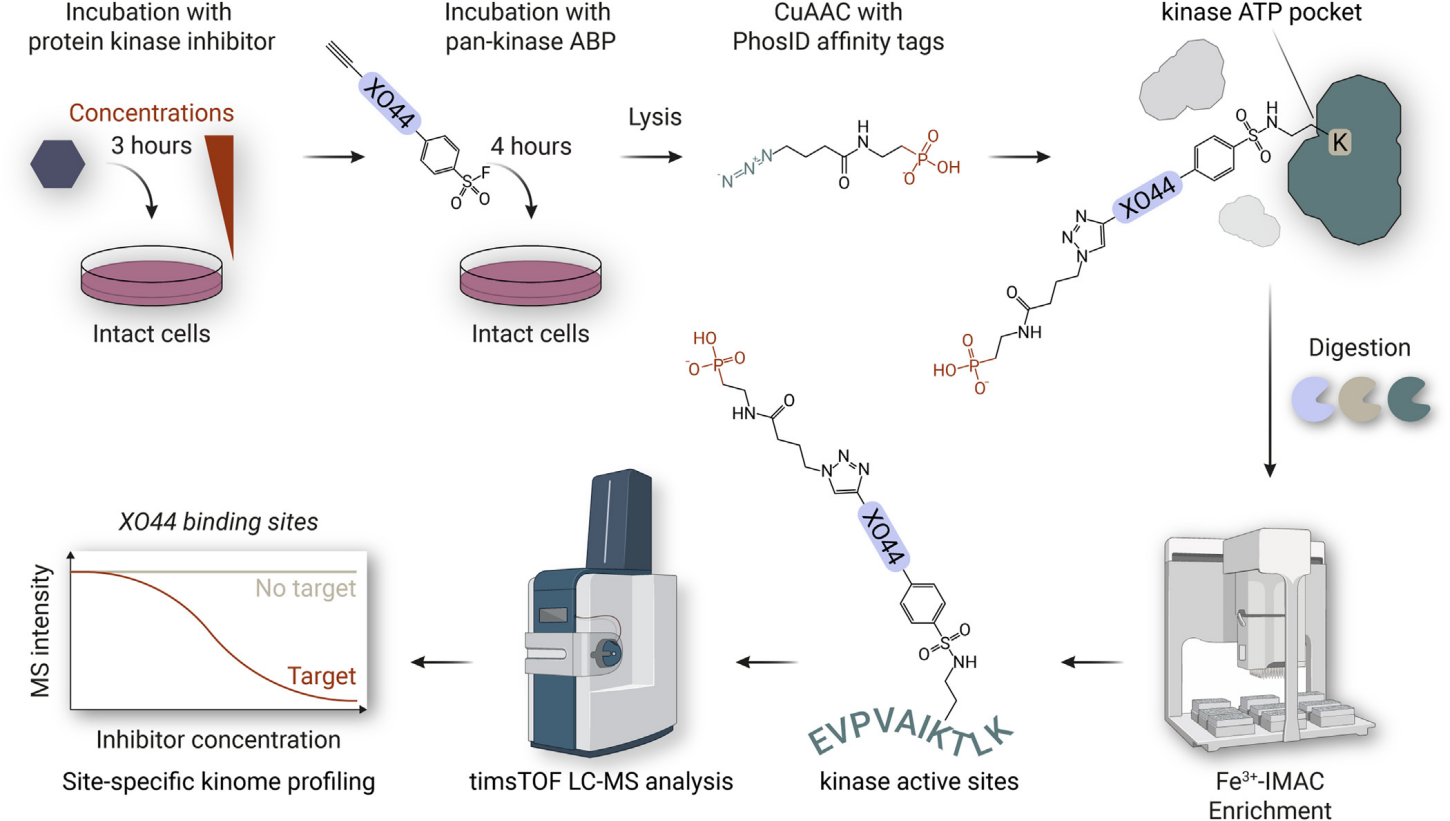
**PhosID-ABPP approach for chemoproteomic site-specific kinome profiling.** Incubation of different inhibitor concentrations and subsequent “chase” with the ABP XO44 in intact cells is followed by cell lysis and attachment of the phosphonate handle by Cu(I)-catalyzed azide–alkyne cycloaddition (CuAAC). Enzymatic digestion and dephosphorylation are performed prior to automated Fe^3+^-IMAC enrichment of XO44-labeled peptides. XO44-labeled peptides are identified and quantified by LC-MS, resulting in the identification of inhibitor targets.

**Fig. 2:**
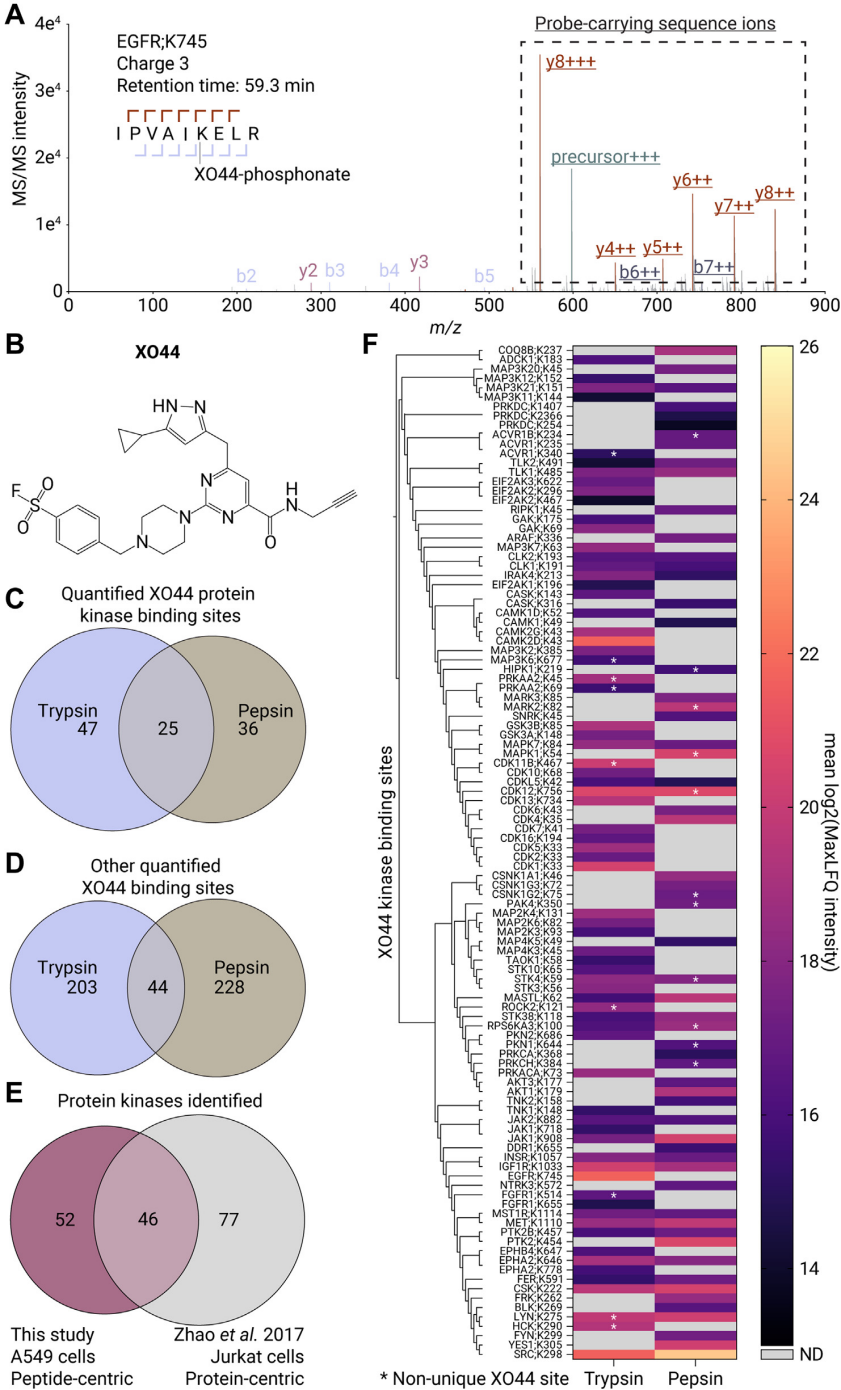
**Mapping XO44 binding sites in A549 cells, using trypsin and pepsin in parallel.***A*, MS/MS spectrum of XO44-bound peptide derived from the ATP pocket in the epidermal growth factor receptor (IPVAIK[XO44-phosphonate]ELR). Underlined ions indicate sequence ions carrying the XO44-phosphonate adduct. *B*, structure of the pan-kinase activity-based probe XO44. *C*, Venn diagram of the overlap of detected XO44-binding sites in protein kinases using trypsin and pepsin. *D*, Venn diagram of the overlap of XO44-binding sites in other proteins, using trypsin and pepsin. *E*, Venn diagram of the detected XO44-bound kinases in this study using a peptide-centric enrichment in A549 cells and the analysis using a protein-centric biotin enrichment in Jurkat cells by Zhao *et al.* (16). *F*, kinome phylogenetic tree of all protein kinases detected and a heatmap displaying the average log2 MaxLFQ intensities of the sum of the XO44-bound peptides corresponding to an XO44-binding site as detected by trypsin- (first column) or pepsin-based (second column) PhosID-ABPP analyses. Asterisks indicate XO44 sites on kinases, albeit only detected through nonunique peptides. ND/*gray* means not detected.

To assess the suitability of XO44 (Figs. 1 and 2*B*) for site-specific PhosID-ABPP, we first performed a noncompetitive analysis using 25 μM of XO44 in intact A549 cells. The strategy, as outlined in Figure 1, using trypsin and omitting the competitive inhibitor, resulted in the successful identification of XO44-labeled lysines in peptides derived from kinase ATP-binding pockets. Identification of these ABP-bound peptides relies on the correct assignment of MS/MS ions derived from sequence ions (b-/y-ions), as well as probe-carrying ions (28). The MS/MS spectrum of the XO44-bound epidermal growth factor receptor’s ATP-binding pocket peptide IPVAIK(XO44-phosphonate)ELR exemplifies the precise identification of ABP-binding sites. The site-specific identification is evidenced by the peptide sequence ions, including those carrying the probe adduct (Fig. 2*A*).

To expand the binding site landscape in site-specific ABPP studies, the use of complementary proteases is essential (28). Moreover, multiple different peptides, generated by different proteases, can provide cumulative evidence for the same drug-binding site. Therefore, we next employed, in parallel, three different proteases (namely trypsin, pepsin, and chymotrypsin) in a pilot experiment, resulting in the identification of 36 XO44-bound protein kinase sites using trypsin, 23 using pepsin, and 12 using chymotrypsin (Supplemental Fig. S1). Trypsin and pepsin exhibited the highest complementarity and captured 93% of the kinases in the combined PhosID-ABPP analysis. Therefore, we chose to employ just these two proteases in parallel for a detailed PhosID-ABPP analysis using XO44.

### Assessing the Site-Specific Kinome Profiling Capabilities of PhosID-ABPP

After selecting the appropriate proteases, we optimized the sensitivity of the PhosID-ABPP method by refining the sample handling process post-enrichment. This involved automated direct elution in an LC-MS–compatible 96-well plate, followed by injecting the entire elution fraction on the LC-MS system. These workflow enhancements significantly improved the detection of XO44-binding sites. Employing pepsin and trypsin in parallel under optimized conditions revealed a total of 1460 XO44-binding sites, including 108 protein kinase sites corresponding to 98 distinct protein kinases (Fig. 2, *B* and *C*). We identified 63 and 50 XO44 kinase-binding sites for trypsin and pepsin, respectively (Fig. 2*B*). Forty six (47%) of the protein kinases were previously detected by Zhao *et al.* using a protein-centric enrichment approach with XO44, albeit that they performed these experiment with Jurkat cells (Fig. 2*E*) (16). Disparities in detected kinases between Zhao *et al.* and our study could be attributed to differences in cell lines, different enrichment strategies, and the use of pepsin as an additional proteolytic enzyme in this study.

The high complementarity of the data generated by using the two proteases was evident, with only 25 overlapping XO44 protein kinase–binding sites detected using both pepsin and trypsin (Fig. 2*B*). Additionally, 44 out of the 475 other lysine XO44-binding sites were shared between pepsin and trypsin (Fig. 2*C*). Both the trypsin- and pepsin-based PhosID-ABPP analytical workflows were highly reproducible, with great overlap between replicates (Supplemental Fig. S2*A*). The R^2^ correlations of ∼0.8 for MaxLFQ intensities of XO44-bound peptides between the replicates indicate reliable quantification (Supplemental Fig. S2*B*) (36). Furthermore, XO44-site MaxLFQ intensities correlated between the proteases for both protein kinases and other proteins (Pearson r correlations of 0.63 and 0.65, respectively, Supplemental Fig. S3).

Phylogenetic classification of the detected kinases highlighted that some groups of evolutionarily conserved kinases were preferentially captured by the same protease. This observation reflects homology in the peptide backbone leading to alike protease cleavage sites proximal to the probe-binding site. Examples include members of the cyclin-dependent kinases family that are preferentially detected using trypsin, and casein kinase family members preferentially detected using pepsin (Fig. 2*F* and Supplemental Fig. S4). However, high homology in kinase-binding sites can also present limitations, as several XO44-bound sites could be mapped to multiple proteins. In our analysis, nine and eleven XO44-bound kinase sites were not unique in the trypsin- or pepsin-assisted PhosID-ABPP analyses, respectively (Fig. 2*F*, annotated with an asterisk). Nevertheless, five of these nonunique sites were uniquely detected by the other protease, including lysine 756 on cyclin-dependent kinase 12 (CDK12;K756), lysine 59 on serine/threonine-protein kinase 4 (STK4;K59), lysine 100 on ribosomal protein S6 kinase alpha-3 (RPS6KA3;K100), lysine 275 on tyrosine-protein kinase Lyn (LYN;K275), underscoring the advantage of using both trypsin and pepsin (Fig. 2*F* and Supplemental Table S1).

Taken together, the site-specific dual-protease kinome profiling, though restricted to XO44 targets, enabled a robust and reproducible identification and quantification of over 100 endogenous kinase-binding sites in intact cells. This success prompted us to further explore competitive protein kinase inhibitor target-profiling capabilities of this platform.

### Site-Specific Proteome-Wide Profiling of NVP-BHG712’s Affinity for Protein Kinases

**Fig. 3:**
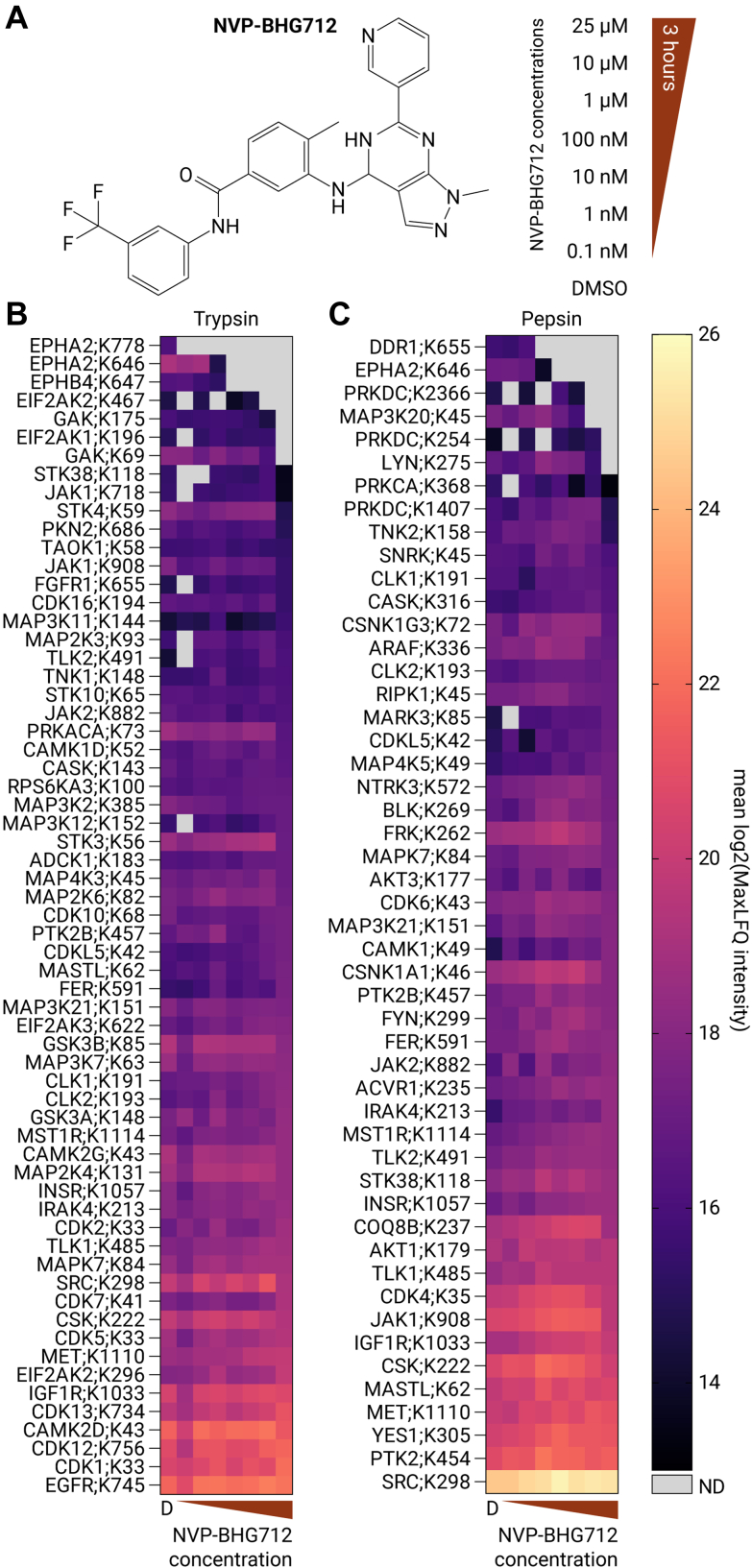
**Competitive dose-dependent profiling of NVP-BHG712.***A*, structure of the ephrin receptor inhibitor, NVP-BHG712, and the inhibitor concentrations used. *B* and *C*, heatmaps displaying the mean log2(MaxLFQ intensity) remaining after treatment with the respective inhibitor concentrations, for each XO44 binding site, using trypsin (*B*) or pepsin (*C*) as the proteolytic enzyme. Supplemental Fig. S5 displays the same data for XO44-binding sites on other protein kinases, albeit only detected by nonunique peptides. D; DMSO, ND, not detected.

Applying the site-specific PhosID-ABPP assay in a competitive setting would allow site-specific drug target screening of virtually any kinase inhibitor against the ABP targets. To assess the capability of the PhosID-ABPP platform in a competitive fashion, we studied the target profile of NVP-BHG712, a noncovalent ephrin receptor B4-targeting inhibitor that has been developed by Novartis to inhibit VEGF-A–mediated angiogenesis in malignancies (Fig. 3*A*) (37, 38). Notably, reversible inhibitor target engagement studies by competitive ABPP are notoriously challenging (39, 40). The competitive setup allowed us to map the site-specific on- and off-target profile of NVP-BHG712 across a range of inhibitor concentrations from low nanomolar to micromolar levels (Fig. 3, *B* and *C*).

**Fig. 4:**
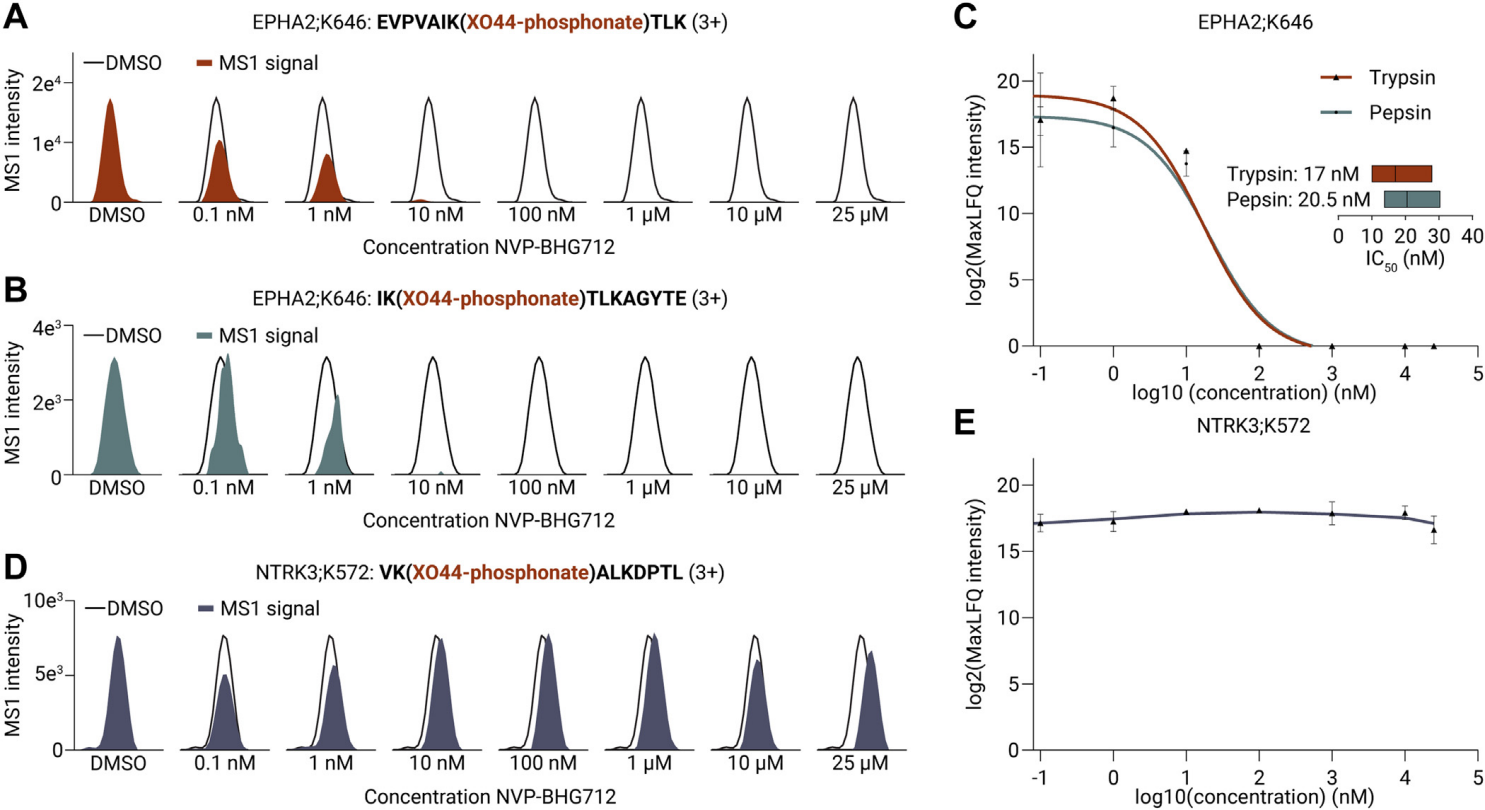
**PhosID-ABPP enables the site-specific dose-response analysis of NVP-BHG712’s target engagement.***A* and *B*, extracted ion chromatograms MS1 intensity of (*A*) a tryptic peptide (EVPVAIK[XO44-phosphonate]TLK, 3+) and (*B*) a peptic peptide (IK[XO44-phosphonate]TLKAGYTE, 3+) reflecting XO44-bound lysine 646 in the ATP pocket on EphA2 across different NVP-BHG712 concentrations. *C*, line plot displaying the log2(MaxLFQ intensity) and the calculated dose-response curve with the IC_50_ values for XO44-bound EPHA2;K646 detected in trypsin and pepsin samples. The insets show the fitted IC_50_ values and the 95% confidence intervals. *D*, extracted ion chromatograms MS1 intensity of a peptic peptide (VK[XO44-phosphonate]ALKDPTL, 3+) reflecting XO44-bound lysine 572 in the ATP pocket on the neurotrophic receptor tyrosine kinase 3 (NTRK3) across different NVP-BHG712 concentrations. *E*, line plot displaying the log2(MaxLFQ intensity) of XO44-bound NTRK3;K572. Error bars indicate the SEM.

Two variants of the Ephrin receptor family, the main target of NVP-BHG712, were detected (Fig. 3, *B* and *C*). Detection of the tryptic peptide EVPVAIK[XO44-phosphonate]TLK (3+) indicated that EphA2 was bound to XO44 *via* lysine 646 within the ATP pocket (Supplemental Fig. S8*A*). Extracted ion chromatography traces of the MS1 intensity for this tryptic peptide, following treatment with different concentrations of NVP-BHG712, revealed a dose-dependent decrease in intensity (Fig. 4*A*). This effect was also observed in a peptic peptide (IK[XO44-phosphonate]TLKAGYTE, 3+) originating from the same binding site (lysine 646) within EphA2 (Fig. 4*B* and Supplemental Fig. S9*A*). After confirming dose-dependent EphA2 inhibition using PhosID-ABPP, the affinity of NVP-BHG712 for EphA2 was determined by analyzing the intensities of all peptides spanning the active site for each protease. The IC_50_ value for EphA2;K646 inhibition by NVP-BHG712 was 17 nM (95% confidence interval [CI] 10–28 nM) for tryptic analysis and 20.5 nM (95% CI 13.7–30.4 nM) for peptic analysis (Fig. 4*C*). These values aligned well with literature (microscale thermo-phoresis: 13 nM; NanoBRET assay: 3 nM; kinobeads: 240 nM) (10). It is important to note that the IC_50_ values obtained through ABPP are apparent and depend on various experimental factors, including the concentration of the target protein and the incubation times of the ABP and the analyzed drug. In addition to EphA2, NVP-BHG712 also bound EphB4 on lysine 647, with an IC_50_ value of 20.2 nM (95% CI: 11.7–33.3 nM) (Supplemental Figs. S6*A* and S8*B*). The equal inhibitory affinities of NVP-BHG712 to EphA2 and EphB4 were consistent with previous data, determined by *in vitro* assays, as well as cellular autophosphorylation of both Ephrin receptors (NVP-BHG712 IC_50_ for autophosphorylation of EphB4: 25 nM) (37). A nonunique XO44-bound peptide that could be derived from the ATP pockets of EphA4 and EphB1 variants, as well as EphA2, showed a similar competitive effect of NVP-BHG712, indicating that NVP-BHG712 might also bind EphA4 and EphB1. Importantly, the signal of the vast majority of other protein kinase XO44 sites signals remained stable over the entire dose range for both trypsin and pepsin (Fig. 3, *B* and *C*). This is exemplified by the extracted ion chromatography traces for the MS1 intensity for the peptic peptide corresponding to the neurotrophic receptor tyrosine kinase 3 (NTRK3) binding site lysine 572 (K572) (Fig. 4*D* and Supplemental Fig. S9*B*). The analysis of the combined intensities for NTRK3;K572 shows a stable MaxLFQ signal over the full concentration range. Similarly, the proto-oncogene tyrosine-protein kinase src (SRC) and the Janus kinases (JAK) 1 and 2 (JAK1;K908 and JAK2;K882) retained a stable signal across the concentration range, showing that these protein kinases do not interact with NVP-BHG712 in their ATP-binding pockets (Supplemental Figs. S6, *B*–*D*, S8, *C*–*E*, and S9, *C*–*E*).

In summary, the site-specific protein kinase inhibitor profiling strategy is robust and reproducible, which is underscored by the highly similar inhibition profiles that were obtained using either trypsin- or pepsin-induced proteolysis. The methodology enabled the calculation of IC_50_ values of the reversible inhibitor NVP-BHG712 for EphA2 and EphB4 in intact cells, and the determined affinities are in line with affinities measured by orthogonal methods.

### The Protein Kinase Profiling Approach Reveals Off-Targets of NVP-BHG712

**Fig. 5:**
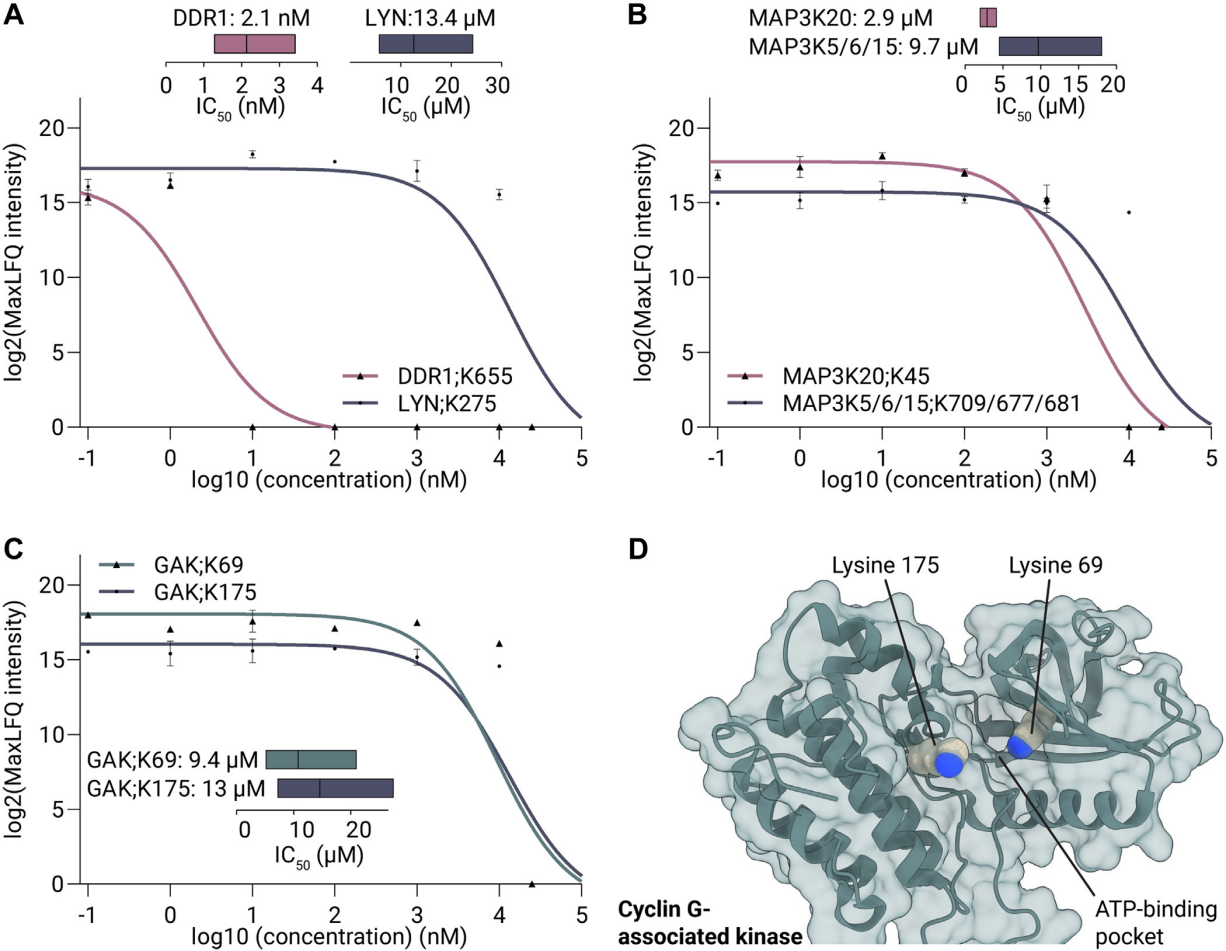
**Dose-response analysis of NVP-BHG712 reveals cancer-relevant protein kinase off-targets.***A*, line plot displaying the log2(MaxLFQ intensity) and the calculated dose-response curve with the IC_50_ values for XO44-bound DDR1;K655 and LYN;K275. GAK;K175, GAK;K69, and MAP3K6;K677. *B*, line plot displaying the log2(MaxLFQ intensity) and the calculated dose-response curve with the IC_50_ values for XO44-bound MAP3K20;K45 and a nonunique peptide belonging to XO44-bound MAP3K5;K709, MAP3K6;K677, and MAP3K15;K681. *C*, line plot displaying the log2(MaxLFQ intensity) and the calculated dose-response curve with the IC_50_ values for XO44-bound lysines 69 and 175 on the cyclin G–associated kinase (GAK). *D*, crystal structure of GAK with ATP pocket lysines 69 and 175 highlighted (PDB:4O38) (44). Error bars indicate the SEM, and the insets show the fitted IC_50_ values with 95% confidence intervals.

Next to the Ephrin receptor family, the pepsin-based PhosID-ABPP analysis revealed competitive NVP-BHG712 binding toward two other tyrosine kinases, the epithelial discoidin domain-containing receptor 1 (DDR1) and LYN on lysine 655 and 275, respectively (Supplemental Fig. S9, *F* and *G*). Calculation of IC_50_ values indicated a strong 2.1 nM (95% CI: 1.3–3.4 nM) affinity of NVP-BHG712 to DDR1, but a much weaker 13.4 μM (95% CI: 6.5–25.1 μM) affinity to LYN (Fig. 5*A*). Intriguingly, recent reports have revealed that several manufacturers sell the regioisomer of NVP-BHG712 (NVP-BHG712-iso) that is slightly different from that described in Novartis’ patent (Supplemental Fig. S7) (10, 38). Profiling both isomers using kinobeads revealed that NVP-BHG712 does not bind to DDR1, whereas NVP-BHG712-iso does (41). Our data clearly reveals strong binding to DDR1, suggesting that the competitive analysis in this study was conducted with NVP-BHG712-iso instead of NVP-BHG712. Recent research on both isomers revealed that NVP-BHG712-iso does not inhibit VEGF-A–dependent angiogenesis while NVP-BHG712 does, underscoring significant phenotypic consequences upon inducing small changes in the chemical structure of small molecule inhibitors (41). Notwithstanding, DDR1 is a relevant drug target in inhibiting colorectal cancer metastasis as well, thus, NVP-BHG712-iso may provide therapeutic opportunities to target this receptor in combination with Ephrin receptors A2 and B4 (42, 43).

A nonunique peptide derived from the ATP pockets of apoptosis signal-regulating kinases 1-3 (MAP3K5/6/15) revealed NVP-BHG712 binding with an IC_50_ of 9.7 μM (95% CI: 4.5–18.1 μM, Fig. 5*B* and Supplemental Figs. S5 and S8*F*). Nonunique peptides present a limitation for peptide-centric ABPP approaches, as they may dilute the effect of the competition, potentially precluding relevant protein kinase inhibitor targets. Furthermore, a unique peptide showed binding of NVP-BHG712 to another MAP3K (MAP3K20;K45) with an IC_50_ of 2.9 μM (95% CI: 1.96–4.2 μM) (Fig. 5*B* and Supplemental Fig. S9*H*). Alongside, XO44 bound to the cyclin G–associated kinase (GAK) on two lysine residues, 69 and 175, both localized within the ATP-binding pocket (Fig. 5*D*, PDB: 4O38, Supplemental Fig. S8, *G* and *H*) (44). Importantly, both sites showed similar IC_50_ values of 9.4 (95% CI: 3.7–19.6 μM) and 13 μM (95% CI: 5.9–26 μM), respectively, emphasizing the robustness of the approach (Fig. 5*C*). While nanomolar affinities are often necessary for drugs to have therapeutic potential, inhibitors with low affinity for cancer-associated targets could provide starting points for optimization toward high-affinity drugs.

### Profiling Non-Kinase Off-Targets of NVP-BHG712

**Fig. 6:**
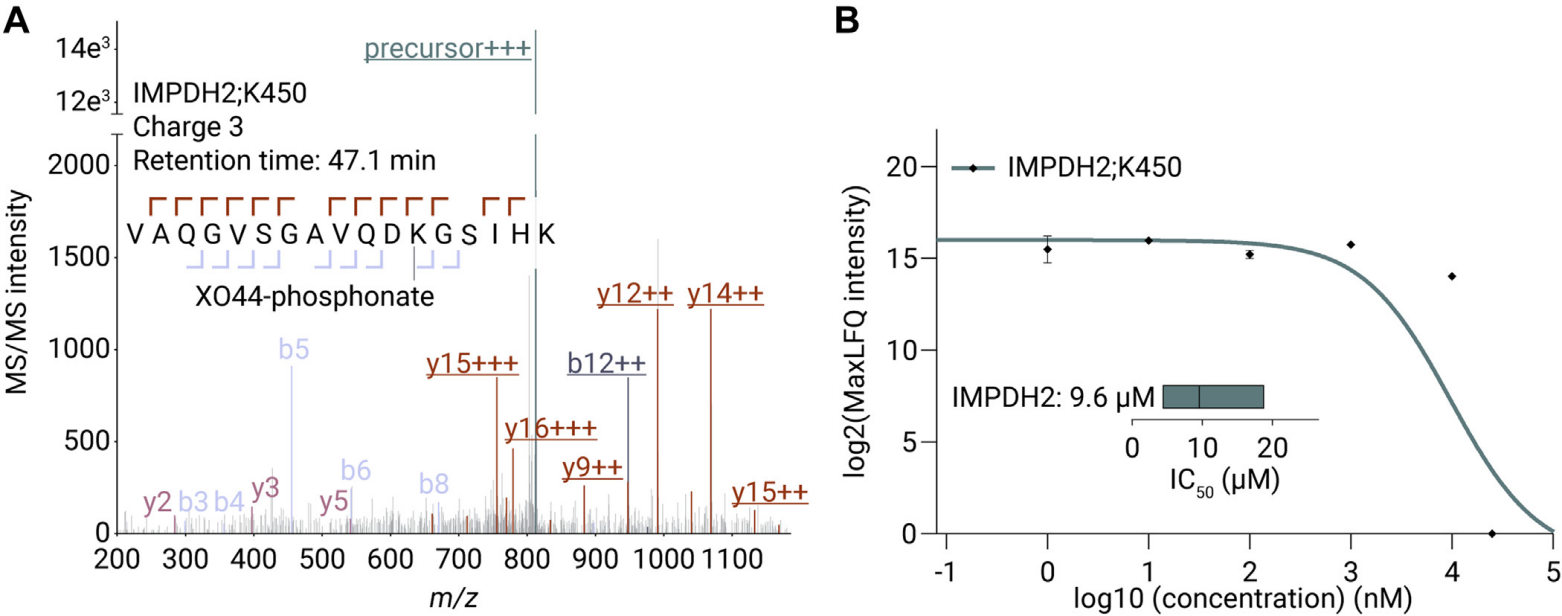
**Non-Kinase off-target binding.***A*, MS/MS spectrum of XO44-bound lysine (450) on a peptide derived from the NAD-binding pocket in inosine-5′-monophosphate dehydrogenase 2 (IMPDH2) peptide VAQGVSGAVQDK[XO44-phosphonate]GSIHK. Underlined ions indicate probe-carrying sequence ions. *B*, line plot displaying the log2(MaxLFQ intensity) and the calculated dose-response curve with the IC_50_ values for XO44-bound IMPDH2;K450. Error bars indicate SD.

The promiscuous binding of XO44 expands the selectivity profiling abilities of the platform beyond proteins belonging to the protein kinase family with an additional 475 proteinaceous lysines. XO44 was found to bind to lysine 450 in the inosine-5′-monophosphate dehydrogenase 2 (IMPDH2, Fig. 6*A*). Moreover, NVP-BHG712 competed with XO44 on this residue with an IC_50_ value of 9.6 μM (95% CI: 4.4–18.8 μM, Fig. 6*B*). Structurally, the lysine is close to the nucleotide-binding pocket in IMPDH2 (45). IMPDH2 plays an essential role in nucleotide metabolism, and its inhibition could potentially result in adverse events (46). As IMPDH2 is also involved in several diseases, including colorectal cancer, inhibition may thus provide therapeutic opportunities as well, with NVP-BHG712 as a potential starting point for drug development (47). Our data emphasize the importance of screening protein kinase inhibitor selectivity beyond protein kinases.

### Advantages of Site-Specific ABPP over Protein-Centric Approaches

Of the 699 proteins that were labeled by XO44, 263 were labeled at multiple sites (Supplemental Fig. S10*A*). The site-specific resolution provides the ability to differentiate inhibition of multiple sites within proteins in parallel. Moreover, by extension of the search parameters for the binding of XO44 to different amino acids within proteins, we identified that the probe also covalently binds to proteinaceous tyrosines (715 sites), as suggested by prior research (27). This significantly expands the scope of the competitive profiling strategy (Supplemental Data S2). For example, seven different XO44-binding sites were detected in the DNA-dependent protein kinase catalytic subunit, which plays a key role in repairing DNA double-strand breaks (Supplemental Fig. S10*B*) (48). The protein was targeted at both lysine and tyrosine residues. The site-specific strategy will allow competitive studies to differentiate and analyze site-specific inhibition by small molecule inhibitors within proteins with multiple binding pockets and active sites.

The site-specific resolution also provides information on the potential functional effect of an inhibitor. For example, labeling of tyrosine 55 in aldo-keto reductase family 1 member C2 was observed; this tyrosine is a proton donor in the active site of this enzyme. Inhibition by XO44 and its competitors at this site are therefore likely to block enzyme activity. Importantly, aldo-keto reductase family 1 member C2 plays a key role in the metabolism of steroids and prostaglandins. Thus, the enzyme is crucial in the regulation of hormonal synthesis and degradation and therefore a potential drug target for modulating dysregulated hormone homeostasis (49).

## Conclusions

Here, we introduce a strategy to profile protein kinase inhibitor selectivity with site-specific resolution. The approach allows screening of inhibitor targets with an amino acid precision of approximately 100 protein kinases and more than 500 other proteins in parallel. The platform was used to assess the target profile of the reversible Ephrin receptor inhibitor NVP-BHG712. Using the site-specific strategy, we found NVP-BHG712 to bind in the ATP pocket of EphA2 and EphB4 with the IC_50_ values of 17 nM (95% CI 10–28 nM) and 20.2 nM (95% CI 13.7–30.4 nM), respectively. These values are in line with previously reported affinities. It is important to note that the IC_50_ values determined by ABPP are apparent IC_50_ values, which are influenced by experimental conditions such as the concentration of the targeted protein and the incubation times of both the ABP and the drug being analyzed. Addition to the Ephrin receptors, NVP-BHG712 bound to the receptor tyrosine kinase DDR1 with an affinity of 2.1 nM (95% CI: 1.3–3.4 nM). Notably, previous research indicated a NVP-BHG712 regioisomer to bind DDR1 with high affinity, but not NVP-BHG712 as patented by Novartis. Several other off-targets of NVP-BHG712, including LYN, apoptosis signal-regulating kinases, MAP3K20, and GAK, were detected, albeit all with micromolar affinity. Binding to GAK was confirmed through binding of XO44 to two lysine residues on two distinct peptides from the same ATP pocket. Furthermore, the promiscuity of XO44 to ATP-binding pockets on other proteins expanded the profiling capabilities of the assay, as our analysis revealed IMPDH2 as an off-target of NVP-BHG712, potentially opening up new therapeutic opportunities. This study used label-free quantification. Future research could focus on the incorporation of isobaric labels to reduce the required protein input and enhance the accuracy of relative quantification.

## Data Availability

The mass spectrometry proteomics data have been deposited to the ProteomeXchange Consortium *via* the PRIDE partner repository with the dataset identifier PXD058749. This article contains supplemental data: Supplemental Figs. S1–S10, Supplemental Table S1, and Supplemental Data Excel files S1 and S2.

## Supplemental data

This article contains supplemental data.

## Conflicts of interest

The authors declare that they have no conflicts of interest with the contents of this article.
