# Supplementary material for: Site-Specific Competitive Kinase Inhibitor Target Profiling Using Phosphonate Affinity Tags
Source: Mol Cell Proteomics. 2025 Jan 16;24(2):100906. doi: 10.1016/j.mcpro.2025.100906 (PMC11889359; doi:10.1016/j.mcpro.2025.100906)
Supplement: Supporting information [file mmc1.pdf]

Supporting information

Site-Specific Competitive Kinase Inhibitor Target Profiling Using Phosphonate Affinity Tags

Wouter van Bergen[1,2], Anneroos E. Nederstigt[1,2], Albert J.R. Heck [1,2], Marc P. Baggelaar[1,2*]

[1] Biomolecular Mass Spectrometry and Proteomics, Bijvoet Center for Biomolecular Research and Utrecht Institute for Pharmaceutical Sciences, University of Utrecht, Padualaan 8, Utrecht 3584 CH, The Netherlands

[2] Netherlands Proteomics Center, Padualaan 8, Utrecht 3584 CH, The Netherlands

[*] Contact details for correspondence: m.p.baggelaar@uu.nl

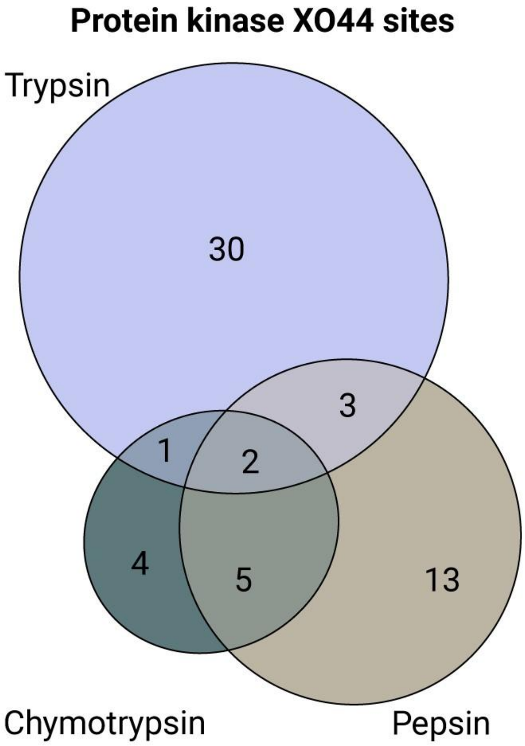

Supplementary Figure 1: Mapping the site-specific XO44 binding sites with trypsin, chymotrypsin, and pepsin in A549 cells. Venn diagram of the overlap in detected XO44 binding sites on protein kinases in PhosID-ABPP analyses using trypsin, chymotrypsin, and pepsin.

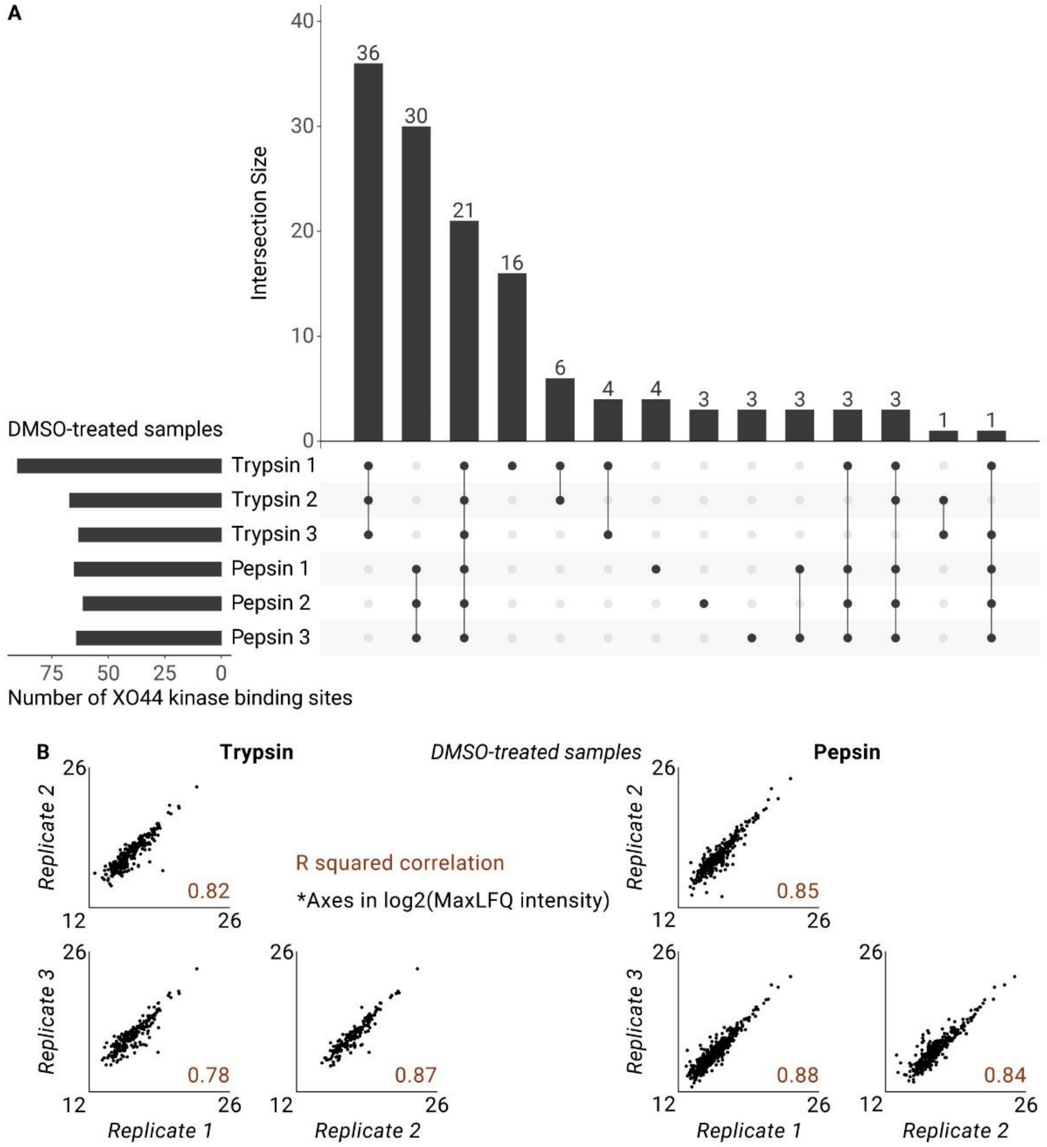

Supplementary Figure 2: A) Upset plot displaying the overlap in XO44 binding sites on kinases in the replicates of PhosID-ABPP analysis using trypsin and pepsin as proteases. Pepsin and trypsin were revealed to be highly complementary. B) Scatter plots showing the correlation of the log2(MaxLFQ intensity) of all XO44-bound peptides over the three replicates of DMSO-treated samples (trypsin (left) and pepsin (right) were used for proteolytic cleavage). The R^2 correlations between the two replicates are annotated in red.

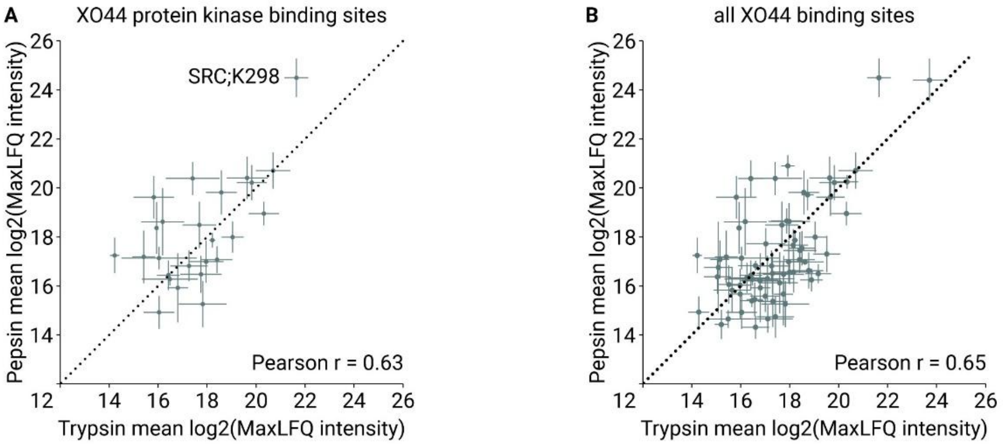

Supplementary Figure 3: A/B) Scatter plot displaying correlation of the mean log2 MaxLFQ intensities of the same XQ44 binding sites detected using both pepsin and trypsin proteolytic workflows. (A) Correlation (Pearson correlation r = 0.63) for XO44 sites in protein kinases detected by trypsin and pepsin. (B) Correlation (Pearson correlation r = 0.65) for XO44 sites in all proteins detected using trypsin and pepsin. Whiskers indicate standard deviation.

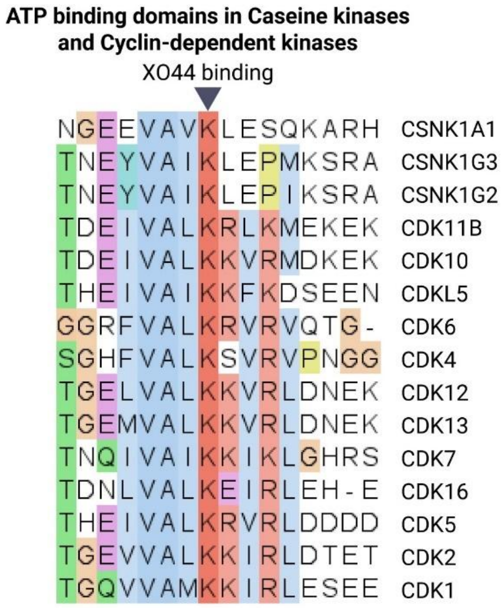

Supplementary Figure 4: Multiple sequence alignment of caseine and cyclin-dependent kinases in the protein sequence surrounding the XO44 binding sites as detected by PhosID-ABPP.

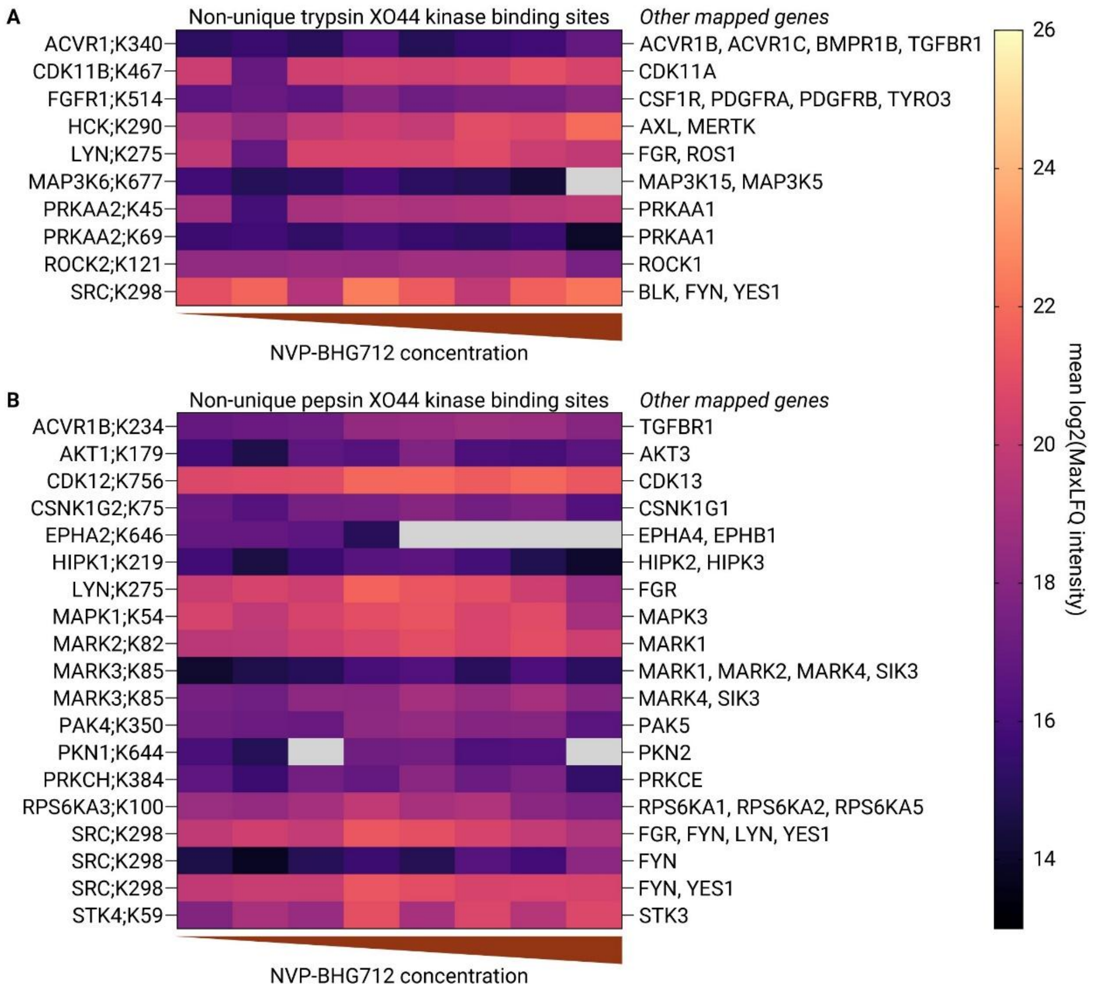

Supplementary Figure 5: Competitive dose-dependent profiling of NVP-BHG712 to non-unique XO44 binding sites. A/B) Heatmaps displaying the mean log2(MaxLFQ intensity) for each non-unique XO44 binding site on kinases using trypsin (A) or pepsin (B) as the proteolytic enzyme. Other mapped genes corresponding to the XO44 binding site are shown on the right of the heatmaps.

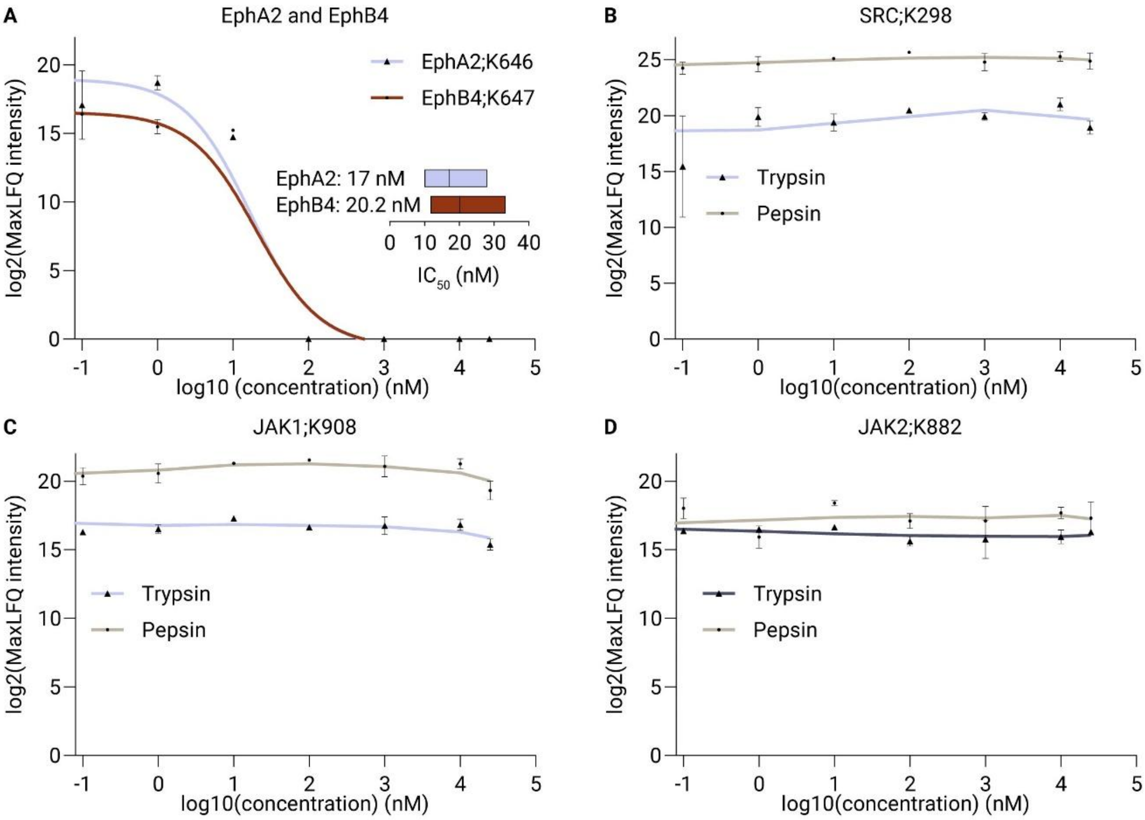

Supplementary Figure 6: A) Line plot displaying the log2(MaxLFQ intensity) and the calculated dose-response curve with the IC_{50} values for XO44-bound EphA2;K646 and EphB4;K647. B-D) Line plots displaying the log2(MaxLFQ intensity) for (B) XO44-bound SRC;K298 and (C) JAK1;K908 and (D) JAK2;K882 detected in trypsin and pepsin samples.

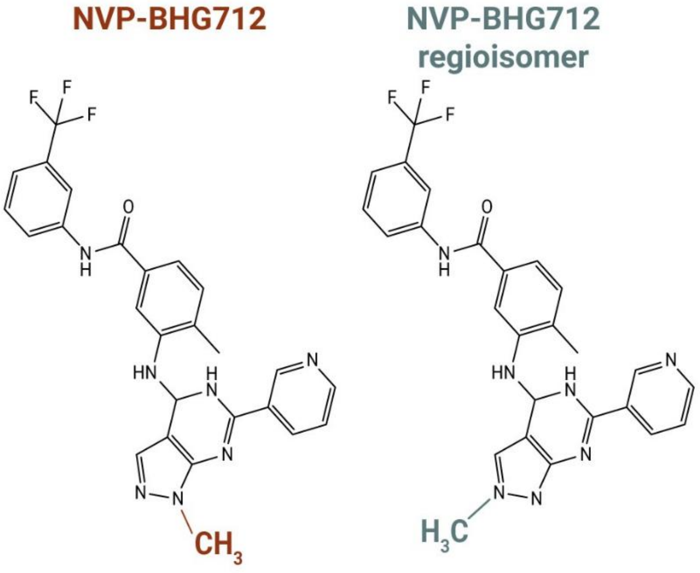

Supplementary Figure 7: Chemical structures of NVP-BHG712 and its regioisomer. The differently positioned methyl groups are highlighted in color.

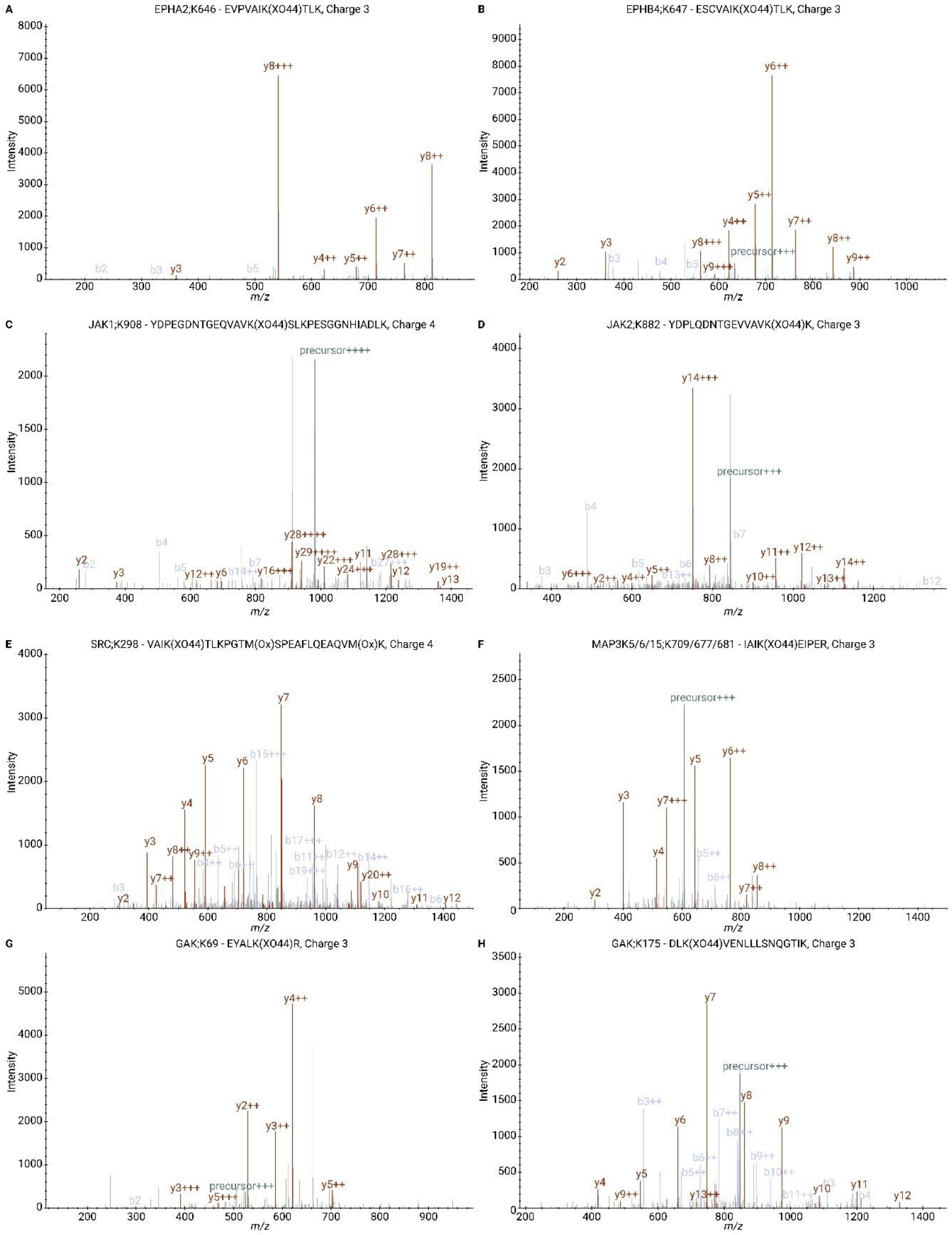

Supplementary Figure 8: Illustrative MS/MS spectra of XO44-bound peptides in trypsin-assisted PhosID-ABPP analysis.

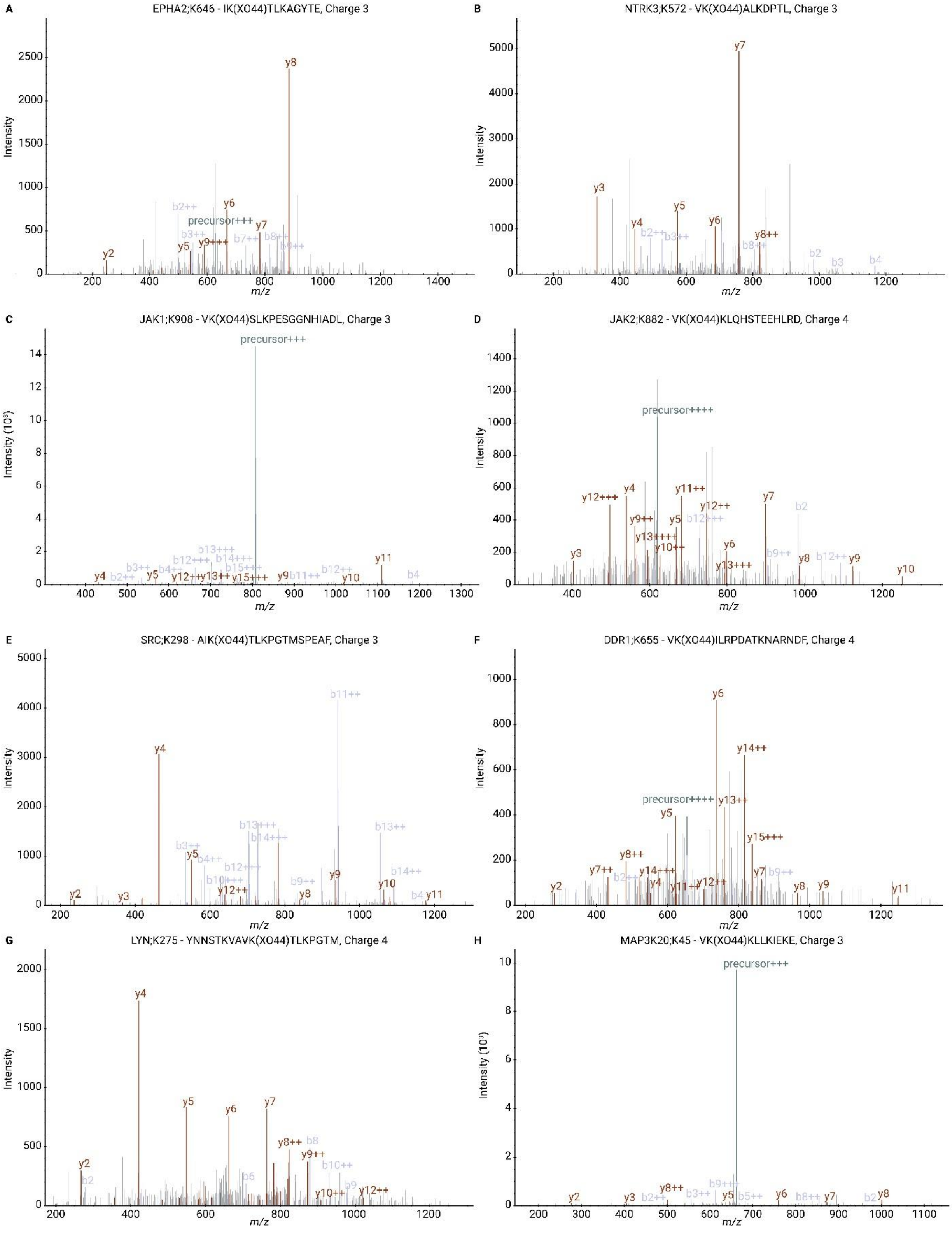

Supplementary Figure 9: Illustrative MS/MS spectra of XO44-bound peptides in pepsin-assisted PhosID-ABPP analysis.

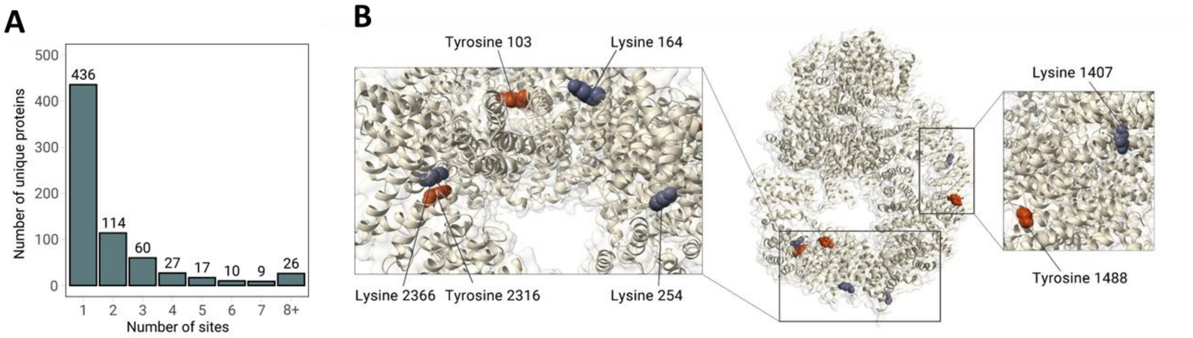

Supplementary Figure 10: A) Overview of the distribution of the number of XO44 binding sites over the detected proteins using the site-specific ABPP strategy. B) Graphical representation of the 7 XO44 binding sites within PRDKC (PDB: 5LUQ).

Supplementary Table 1: Non-unique XO44 binding sites in trypsin or pepsin-based PhosID-ABPP analyses that are uniquely identified by the other protease.

**XO44 binding site**	***Trypsin***		***Pepsin***		**Other mapped genes**
	**Unique**	**Peptide**	**Unique**	**Peptide**	
CDK12;K756	Y	DKDTGELVALK(XO44)K	N	VALK(XO44)KVRLDNEKEGFPITA	CDK13
STK4;K59	Y	ETGQIVAIK(XO44)QVPVESDLQEIIK	N	IK(XO44)QVPVESD	STK3
RPS6KA3:K100	Y	QLYAMK(XO44)VLK	N	MK(XO44)VLKKATI	RPS6KA1/2/5
LYN;K275	N	VAVK(XO44)TLK	Y	YNNSTKVAVK(XO44)TLKPGTM	FGR, ROS1
